# Preliminary Assessment of Polysaccharide-Based Emulgels Containing Delta-Aminolevulinic Acid for Oral *Lichen planus* Treatment

**DOI:** 10.3390/ph16111534

**Published:** 2023-10-30

**Authors:** Emilia Szymańska, Joanna Potaś, Mateusz Maciejczyk, Magdalena Ewa Sulewska, Małgorzata Pietruska, Anna Zalewska, Aleksandra Pietruska, Katarzyna Winnicka

**Affiliations:** 1 Department of Pharmaceutical Technology, Medical University of Białystok, Mickiewicza 2c, 15-222 Białystok, Poland; joanna.potas@umb.edu.pl (J.P.); katarzyna.winnicka@umb.edu.pl (K.W.); 2 Department of Hygiene and Epidemiology, Medical University of Białystok, Mickiewicza 2c, 15-222 Białystok, Poland; mateusz.maciejczyk@umb.edu.pl; 3 Department of Periodontal and Oral Mucosa Diseases, Medical University of Białystok, Waszyngtona 13, 15-269 Białystok, Poland; magdalena.sulewska@umb.edu.pl (M.E.S.); mpietruska@wp.pl (M.P.); 4 Independent Laboratory of Experimental Dentistry, Restorative Dentistry Department, Medical University of Białystok, Waszyngtona 13, 15-269 Białystok, Poland; azalewska426@gmail.com; 5 Student’s Research Group, Department of Periodontal and Oral Mucosa Diseases, Medical University of Białystok, Waszyngtona 13, 15-269 Białystok, Poland; aleksandra.pietruska@gmail.com

**Keywords:** tragacanth, xanthan, gellan gum, oral lichen planus, delta-aminolevulinic acid, oromucosal delivery, emulgel

## Abstract

Photodynamic therapy using delta-aminolevulinic acid is considered a promising option in the treatment of oral lichen planus. In the present work, three emulgel compositions prepared from natural polysaccharide gums, tragacanth, xanthan and gellan, were preliminarily tested for oromucosal delivery of delta-aminolevulinic acid. Apart from cytotoxicity studies in two gingival cell lines, the precise goal was to investigate whether the presence of the drug altered the rheological and mucoadhesive behavior of applied gelling agents and to examine how dilution with saliva fluid influenced the retention of the designed emulgels by oromucosal tissue. Ex vivo mucoadhesive studies revealed that a combination of xanthan and gellan gum enhanced carrier retention by buccal tissue even upon dilution with the saliva. In turn, the incorporation of delta-aminolevulinic acid favored interactions with mucosal tissue, particularly formulations comprised of tragacanth. The designed preparations had no significant impact on the cell viability after a 24 h incubation in the tested concentration range. Cytotoxicity studies demonstrated that tragacanth-based and gellan/xanthan-based emulgels might exert a protective effect on the metabolic activity of human gingival fibroblasts and keratinocytes. Overall, the presented data show the potential of designed emulgels as oromucosal platforms for delta-aminolevulinic acid delivery.

## 1. Introduction

Oral lichen planus (OLP) is an inflammatory disease of the mucus membranes in the oral cavity, including the buccal mucosa, tongue and the gingiva [1]. OLP affects 1–2% of the population, in particular middle-aged people of 30–60 years of age, more frequently females. Although the exact etiology is not fully understand, it is linked to an autoimmune response wherein cytotoxic T-cells initiate apoptosis of the basal epithelial cells [1,2]. OLP may be activated by several factors, including chronic diseases (bowel, diabetes, cancer), systemic drugs, infectious agents (in particular hepatitis C virus and H. pylori), or certain types of dental materials, e.g., silver amalgam [1].

Basically, there are two approaches for OLP treatment, the surgical and the non-invasive procedure [3]. Surgical methods include excision, electrocauterization, cryosurgery and carbon dioxide laser ablation. Non-surgical treatment comprises either topical or systemic administration of glucocorticoids, immunomodulators, or systemic immunosuppressants. In recent years, photodynamic therapy (PDT) has been applied as a non-invasive therapeutic option for the management of OLP [4,5,6]. The fundamental elements of PDT include a photosensitizer which is excited by visible light at a specific wavelength. At present, the clinically approved photosensitizer is 5-aminolevulinic acid hydrochloride (ALA) [7]. ALA, a water-soluble molecule, belongs to the class of alpha-amino ketones. It is a natural precursor involved in the synthesis pathway of endogenous protoporphyrin IX. Subsequent activation by infrared light leads to the formation of reactive singlet oxygen, responsible for irreversible damage of targeted cells by a complex cascade of biological and chemical reactions [8,9]. ALA-PDT is considered a selective, well tolerated and minimally invasive therapeutic option with a low risk of damage to underlying functional tissues [7,10]. 

The present studies aimed at developing ALA-loaded emulgels as oromucosal delivery platforms capable of providing prolonged contact between the drug and oral mucosa. Emulgels, being semisolid formulations, appear in convenient dosage forms for oromucosal administration due to ease of application and good spreadability over the topical lesions [11]. Nonetheless, a challenge in effective oromucosal drug delivery is to ensure its prolonged retention to oral mucosa in spite of constant saliva flushing. This is particularly important in terms of ALA delivery, as it requires sufficient time to stay in contact with the tissue before exposition to the light [12]. Therefore, mucoadhesive, biodegradable polymers, namely xanthan (XA), tragacanth (TG) and gellan gums (GG) [13,14,15,16], were selected for development studies of an oromucosal ALA delivery system. Prepared emulgels were characterized in terms of rheological studies and ex vivo mucoretention measurements using porcine buccal epithelium. This precise effort was made to investigate whether the presence of ALA altered the rheological and mucoadhesive behavior of applied gelling agents and to examine how dilution with saliva influenced the retention of emulgels by oromucosal tissue. Furthermore, this study focused on the cytotoxicity examination of ALA-loaded emulgels by MTT assay followed by a mitochondrial membrane potential test and an Annexin V binding assay in human gingival fibroblasts and keratinocytes. 

## 2. Results and Discussion

### 2.1. Rheological Behavior and Mucoretention Characteristics

The application of ALA-PDT as a therapeutic option for OLP has attracted great attention in recent years. Despite comprehensive data providing evidence of its efficacy toward oral premalignant diseases, including OLP, leukoplakia, or erythroplakia [4,5,17], at present there is no commercially available ALA product for oral delivery. The key solution may lie in the development of an oromucosal formulation with the ability to prolong drug retention by mucosal lesions to improve ALA efficacy. In these studies, three emulgels based on natural polymer excipients, namely TG in combination with XA (F1), TG (F2), and GG in combination with XA (F3), were developed and preliminary tested in terms of their oromucosal applicability, measured by rheological and mucoadhesive characteristics. Due to insufficient consistency and poor applicability, the formulation comprising solely GG was not considered in the studies. Prepared ALA-loaded formulations F1–F3 displayed smooth, uniform consistency with pH values ranging from 3.7 to 3.9. The drug was uniformly dispersed within the emulgel base, fulfilling the acceptable limit of 90–110% [18]. 

**Figure 1:**
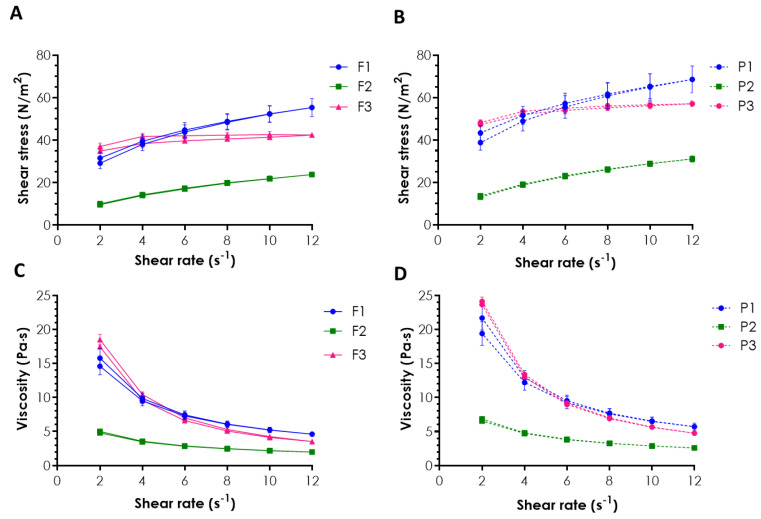
Rheograms (**A**,**B**) and plots of viscosity vs. shear rate (**C**,**D**) of emulgels containing ALA (**A**,**C**) and corresponding placebo formulations (**B**,**D**) diluted with simulated saliva fluid at the weight ratio of 1:0.5 assessed at 37 ± 1 °C (mean ± SD, *n* = 3).

The rheological measurements were performed at 37 ± 1 °C upon the samples’ dilution in a ratio of 1:0.5 (*w*/*w*) with SSF (pH 6.8) to simulate the environment within the oral cavity and the changes that might occur after application. The rheological behavior of drug-free and ALA-loaded oromucosal preparations is shown in Figure 1. 

In the rheograms, a drop in viscosity values with an increasing shear rate was noticed for all tested emulgels (Figure 1C,D), indicating their non-Newtonian shear-thinning properties. Preparations with pseudoplastic behavior are considered appropriate for mucosal application, as they offer improved flow and help to maintain the drug at the mucosal tissue [11,19]. Basically, formulation TG/XA (F1) and formulation GG/XA (F3) (together with their placebo counterparts P1 and P3) showed comparable viscosity upon growing the shear rate. In turn, compositions F2 and P2 (using TG as the only viscosity-enhancing agent) were found to be profoundly less viscous. The presence of the drug was responsible for a slight drop in the initial viscosity values of emulgels F1–F3 vs. their placebo counterparts but did not affect the overall rheological behavior of the tested formulations (Figure 1A vs. Figure 1B, and Figure 1C vs. Figure 1D). In addition, formulations F1 and F3 possessed thixotropic properties, as demonstrated by the presence of a hysteresis loop, a representative shift of the lower curve in comparison to the upper one (Figure 1A,B). The observed loss of viscosity upon mechanical stress facilitates the formulation’s removal from the container and its spreading all over the tissue. In turn, fast structure recovery upon application favors drug retention at the application site. The observed difference in rheograms (Figure 1A,B) indicated that the presence of XA in emulgels F1 and F3 favored their ability for gradual recovery after removing the shear stress when compared to TG-based formulation F2. A lack of thixotropic properties, noticed for F2, may impair the final applicability and retentivity upon oromucosal application [20].

**Figure 2:**
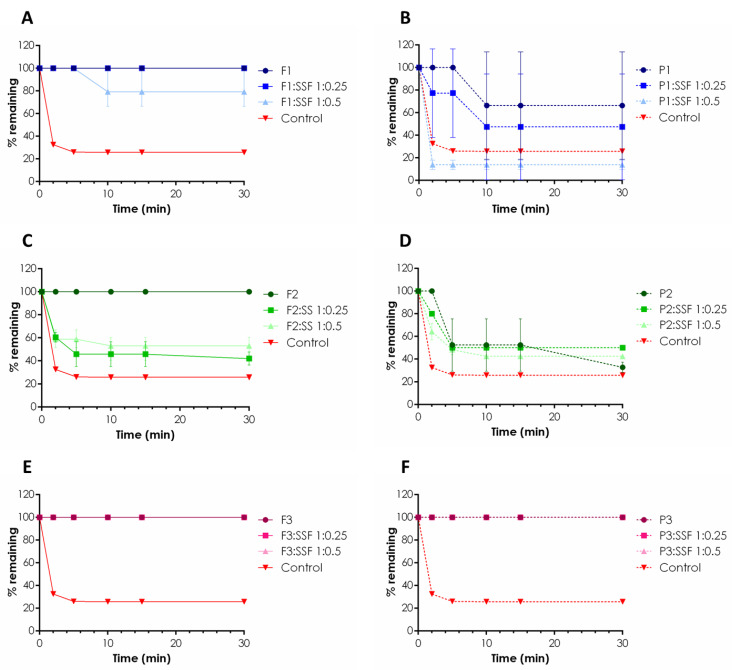
In vitro mucoretention profile of ALA-loaded emulgels (F1–F3) (**A**,**C**,**E**) and placebo (P1–P3) (**B**,**D**,**F**) expressed as % of weight of undiluted emulgel or formulation diluted with SSF in the ratio of 1:0.25 or 1:0.5 that remained on the porcine buccal mucosa; water was used as control (mean ± SD, *n* = 3).

Emulgels were, next, applied to a mucoretention assay to examine and compare their ability to interact with porcine buccal mucosa using a gravimetric technique according to Sandri et al. [21]. Despite the fact that TG, XA and GG are considered mucoadhesive excipients with great potential in the technology of oral drug preparations [22], hardly any data exist on their mucoadhesive behavior when in contact with saliva. The constant flow of saliva and the risk of drug swallowing are considered the main factors impeding oromucosal formulations’ performance after application. Therefore, the idea was to investigate how dilution with saliva influenced the retention of designed emulgels F1–F3 by the mucosal tissue. For this purpose, emulgels were tested in an undiluted state and upon dilution with SSF in weight ratios 1:0.25 and 1:0.5, the conditions imitating a gradual dilution of the formulation occurring in the oral cavity. The results are displayed in Figure 2.

Basically, all ALA-loaded formulations, F1–F3, tested in an undiluted state were capable of being retained on the buccal epithelium without any weight loss through the study period (Figure 2A,C,E). In contrast, control studies with water indicated about 70% and 80% weight losses after the first 2 and 5 min of the test (Figure 2). Some differences in mucoretention behavior occurred upon sample dilution with SSF (pH 6.8). Emulgel F1 with TG/XA, when diluted with SSF in a weight ratio 1:0.5, flew down gradually from the buccal tissue segment, and about a 20% sample loss after 10 min was noticed. It should be emphasized that this formulation was capable of remaining entirely on the tissue surface when diluted with SSF in a ratio 1:0.25 (Figure 2A). When comparing formulations F1 and F2, the presence of XA appeared to prolong the contact of the TG-based composition with the oromucosal tissue. Emulgel F2 was found to be profoundly less adhesive, and its gradual removal from inclined porcine buccal tissue was observed with about a 40–60% loss in mucoretention values upon dilutions 1:0.25 and 1:0.5 (*w*/*w*), respectively (Figure 2C). That probably can be attributed to its weaker rheological properties and lower viscosity values when compared to those attained for emulgel F1 (Figure 1). Interestingly, emulgel F3 remained on the tissue surface in all tested variants of dilution, suggesting the composition prepared of GG and XA is more resilient to tensions appearing within the oral cavity, even upon wetting with the saliva. Some differences in mucoadhesive properties were observed in the studies with drug-free emulgels, and the presence of ALA was found to influence the retention behavior, particularly formulations comprising TG. In fact, undiluted placebo emulgels P1 and P2 were not able to retain on the tissue surface, and about 30% and 70% sample losses within the first 10 min were noticed, respectively. In turn, in studies with ALA-loaded formulations, the incorporation of the drug appeared to support and enhance interactions of the TG/XA (F1) (Figure 2A,B) and TG compositions (F2) with mucosal tissue (Figure 2C,D). In general, the presence of drug particles within a polymeric drug carrier is considered a factor responsible for a drop in mucoadhesiveness, e.g., by reducing its effective adhesion surface area [23,24]. ALA may have acted as plasticizer, but, only in the case of TG-based compositions, that was by increasing TG chains’ flexibility, improving the hydrogen bonding potential with mucosal tissue [25,26]. In turn, incorporation of ALA into the GG/XA base did not modify the level of polymer chains’ entanglement, as emulgel P3 performed with comparable mucoadhesiveness to its ALA-loaded counterpart, and no real impact of the drug presence on the mucoretention profile of the GG/XA formulation was noticed. Overall, the combination of GG and XA appeared to enhance carrier retention to buccal tissue even upon dilution with the saliva. Formulation F3 exhibited the most favorable mucoretention profile, which should prolong the contact of photosensitizer with tissue lesions prior to light excitation. 

### 2.2. Cytotoxicity Profile

**Figure 3:**
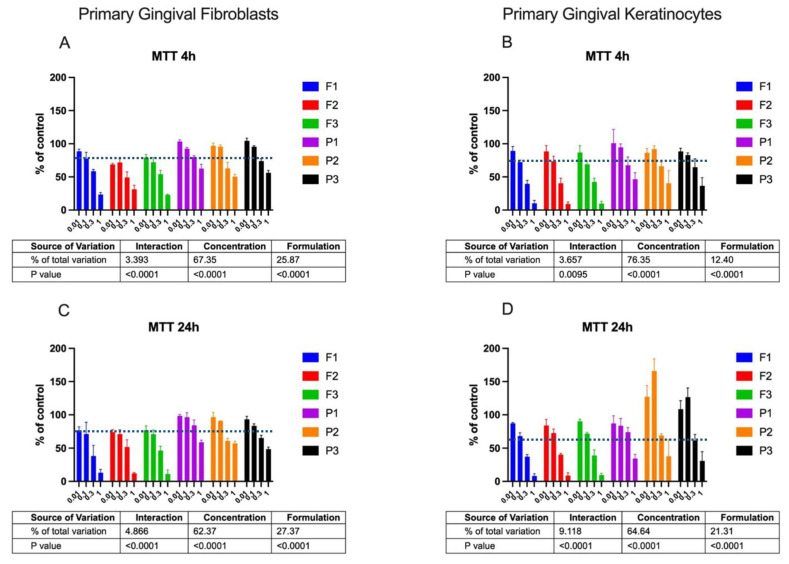
MTT viability (expressed as % of control) of gingival fibroblasts (**A**,**C**) or gingival keratinocytes (**B**,**D**) after 4 h (**A**,**B**) or 24 h (**C**,**D**) incubation with emulgels containing delta-aminolevulinic acid (F1–F3) and corresponding placebo formulations (P1–P3) in concentration range 0.01–1.0 mg/mL in comparison to untreated cells (100%) and pure ALA (blue line) (mean ± S.D.; *n* = 4).

The intimate contact between the mucoadhesive drug carriers and the oromucosal tissue requires formulations to be nonirritant and low-toxic. The preliminary assessment of the cytotoxicity profile of the designed emulgels was carried out in two human cell lines: primary gingival fibroblasts and keratinocytes. The effects of drug-free and ALA-loaded formulations in the concentration range 0.01–1.0 mg/mL on the cell metabolic activity measured by MTT assay is shown in Figure 3.

Basically, all tested ALA-loaded formulations did not affect the metabolic activity of fibroblasts and keratinocytes over 24 h at a concentration of 0.01 mg/mL. When compared to untreated cells, a slight decrease in fibroblast viability (up to 71.0–71.4%) and keratinocyte viability (up to 74.2–78.0%) was observed after 24 h of incubation with formulations at 0.1 mg/mL. In turn, the highest tested concentration, 1 mg/mL, reduced cell viability below 30% after 4 h and below 15% after 24 h. No significant differences among designed compositions F1–F3 on the metabolic activity of both tested cell lines were noticed. The MTT test displayed significant variations in mitochondrial functions between cells exposed to drug-loaded and placebo groups (*p* < 0.0001), and ALA-loaded formulations exhibited greater inhibitory effect on fibroblast and keratinocyte viability. This is in agreement with control studies using ALA, wherein the clear impact of the pure drug on the cell viability was noted, with a drop in cell metabolic activity by up to 73 ± 5% after 4 h and 67 ± 6% after 24 h. The observed effect exerted by the drug may be attributed to its acidic behavior causing a decrease in pH of the culture medium, which in turn may have initiated the cell response.

Interestingly, a substantial increase in metabolically active gingival keratinocytes was observed after incubation with placebo formulations at concentrations of 0.01 and 0.1 mg/mL. This positive impact on the keratinocytes’ growth and an increase in their metabolic activity was particularly visible in cells incubated with formulations P2 and P3. All tested emulgels exhibited time-dependent cytotoxicity when applied in the highest tested concentration. It should be noted that the MTT assay detected reduced metabolic activity but no cell death itself. The observed cytotoxic effect may, therefore, be unspecific and related, rather, to mechanical stress exerted on the cell monolayer by the higher osmolality and overall concentration of formulation ingredients.

**Figure 4:**
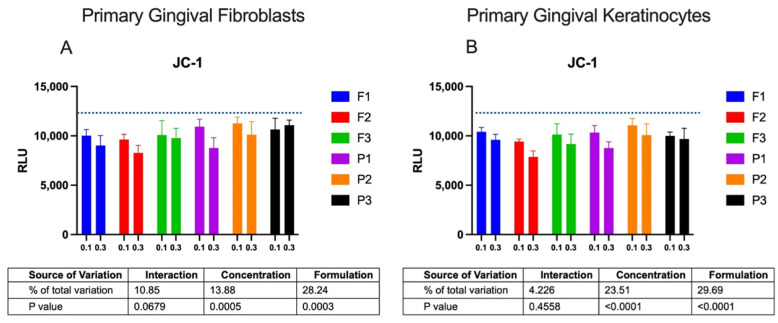
Intensity of luminescence (RLU)) displaying the mitochondrial membrane potential (ΔΨM) of gingival fibroblasts (**A**) or keratinocytes (**B**) after 24 h incubation with 0.1 or 0.3 mg/mL of drug-free or ALA-loaded emulgels measured by JC-1 assay in comparison to untreated cells (blue line); RLUs of cells treated with pure drug (ALA) at concentration corresponding to those encapsulated in 0.3 mg/mL drug-loaded formulations were 8832 ± 954 and 8644 ± 1375 for fibroblasts and keratinocytes, respectively (mean ± S.D.; *n* = 4).

Cytotoxicity was next tested by a JC-1 assay that detects the loss of ΔΨM. The effect of ALA-loaded and placebo formulations on the ΔΨM of gingival cells after 24 h of incubation is shown in Figure 4. Based on the MTT data, two concentrations, 0.1 and 0.3 mg/mL, of drug-free and ALA-loaded formulations were applied in tests referring to their plausible sub-toxic concentrations. 

Compared to the spontaneous loss in ΔΨM observed in control cells, gingival fibroblasts and keratinocytes incubated with emulgels displayed some decrease in the level of luminescence. Basically, the amount of cells with the drop in ΔΨM increased with a rise in the sample’s concentration from 0.1 to 0.3 mg/mL (*p* < 0.0005 and *p* < 0.0001 for fibroblasts and keratinocytes, respectively). It should be noted that despite tested emulgels causing a loss in mitochondrial membrane integrity after a 24 h incubation, the drop in ΔΨM did not exceed 15–20% in any of the tested formulations, except for tests with emulgel F2, where about a 30% loss in ΔΨM was noticed. A lower intensity of luminescence was found for cells exposed to drug-free formulations P1–P3 (*p* = 0.0003 and *p* < 0.0001 for fibroblasts and keratinocytes, respectively), which correlated with control data attained for pure ALA incubated with cells and indicated the cytotoxic potential of the encapsulated drug. The JC-1 assay revealed no effect of drug-free formulations P2 and P3 on the ΔΨM of fibroblasts and keratinocyte cells upon a 24 h exposure, which supported data from the MTT assay and demonstrated that TG-based and GG/XA-based formulations might possess protective effects toward the metabolic activity of gingival cells. 

**Figure 5:**
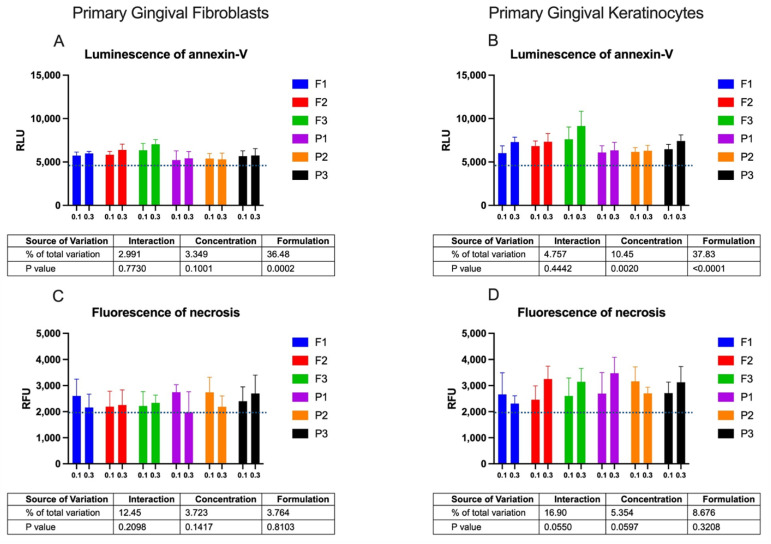
Evaluation of apoptosis measured by intensity of luminescence (**A**,**B**) and necrosis level (**C**,**D**) of gingival fibroblasts (**A**,**C**) or gingival keratinocytes (**B**,**D**) after 24 h incubation with emulgels containing delta-aminolevulinic acid (F1–F3) and corresponding placebo formulations (P1–P3) in concentrations 0.1 and 0.3 mg/mL in comparison to untreated cells (blue line); for the positive control (50 mM H_2_O_2_), RLU was 964,542 ± 32,319 and RFU was 35,750 ± 2511; for pure drug (ALA) RLU was 6585 ± 293 and RFU was 2513 ± 350 (mean ± S.D.; *n* = 4).

Cytotoxicity was additionally tested by an apoptosis test to distinguish the type of cell death. The Real-Time–Glo™ Annexin V Assay, by measuring the exposure of phosphatidylserine on the outer surface of the cell membrane, detected cells that have entered apoptosis or necrosis processes. The binding of Annexin V (apoptosis) was assessed with a luminescence signal, whereas necrosis was assessed with a fluorescence signal. The amount of apoptotic and necrotic cells after 24 h incubation with drug-free or ALA-loaded emulgels is presented in Figure 5. 

The 24 h incubation of gingival cells with emulgels at concentrations 0.1 and 0.3 mg/mL caused low exposure of phosphatidylserine when compared to untreated cells (Figure 5A,B). The apoptotic effect of drug-loaded formulations was stronger than that evoked by placebo samples. A greater intensity of luminescence signals for drug-loaded formulations F1–F3 was particularly visible in tests with the keratinocyte cell line. Notably, all tested formulations, F1–F3, exerted negligible effects on inducing apoptosis in comparison with the positive control, which displayed two orders of magnitude greater values of luminescence upon 24 h of incubation. Similarly, a relatively low level of the fluorescence signal was noted for the tested emulgels when compared to the positive control, indicating that no real necrotic effect was involved upon a 24 h incubation with designed formulations (Figure 5C,D). Overall, results from cytotoxicity tests showed that different combinations of tested mucoadhesive agents in designed formulations had no significant impact on gingival cell viability upon 24 h incubation in the concentration range 0.01–0.3 mg/mL. Incubation with the highest tested concentration of ALA-loaded samples initiated programmed cell death mediated by a mitochondrial pathway. Further, more detailed studies will evaluate the safety profile of designed ALA-loaded emulgels in contact with soft oral tissues. Importantly, the presented findings enabled the selection of the concentration range 0.01–0.3 mg/mL as safe and nonirritant in cell cultures; this can help to estimate dosing and time schedules for toxicology studies. 

## 3. Materials and Methods

### 3.1. Materials

TG from *Astragalus gummifer*, composed primarily of tragacanthin and bassorin, with average viscosity of 1%, aqueous dispersion 200 cPas at 25 °C and XA (average viscosity of 1% aqueous dispersion 1500 cPas at 25 °C) was purchased from Sigma-Aldrich (St. Louis, MO, USA). High acyl GG (Kelcogel CG-HA) was obtained from CP Kelco (Atlanta, GA, USA). Delta-aminolevulinic acid hydrochloride (ALA, purity ≥ 99%, serial number 19022020) was purchased from Syntal Chemicals Sp. z o.o. (Gliwice, Poland). *Soybean phosphatidylcholine* was from Lipoid (Kőln, Germany) and propylene glycol was obtained from Avantor Performance (Gliwice, Poland). Castor oil (pharmaceutical grade) was purchased from Coel (Kraków, Poland). *Disodium* dihydrogen ethylenediaminetetraacetate and sodium benzoate were from ChemPur (Piekary Śląskie, Poland). Fetal bovine serum (FBS), Dulbecco’s modified Eagle medium (DMEM), phosphate-buffered saline (PBS, pH 7.0) and trypsin were purchased from Gibco (Gaithersburg, MD, USA). Penicillin, streptomycin, 3-(4,5-dimethylthiazol-2-yl)-2,5-diphenyltetrazolium bromide (MTT), dimethyl sulfoxide (DMSO), glycine, sodium chloride, scintillation fluid Ultima Gold XR and Hydrogen peroxide (H_2_O_2_) were purchased from Sigma Aldrich (St Louis, MO, USA or Steinheim, Germany). The simulated saliva fluid (SSF) with composition 0.1 M disodium hydrogen phosphate and 0.1 M potassium dihydrogen phosphate (ChemPur, Piekary Śląskie, Poland) was prepared according to [27] and adjusted to pH 6.8 with sodium hydroxide. Commercially available porcine cheek tissue applied in mucoretention studies was obtained from the veterinary service of local slaughterhouse (Turośń Kościelna, Poland).

### 3.2. Emulgel Preparation and Characterization

Emulgels were prepared by homogenization technique using TG, XA or GG. Briefly, TG in combination with XA (formulation F1) or TG solely (formulation F2) was gradually dispersed in water (in weight ratio 6.0/86.3 for F1 and 5.0/87.3 for F2) and homogenized at 1400 rpm in automatic homogenizing system (Unguator E/S Eprus, Poland). For formulation F3, GG was dispersed (polymer to water weight ratio 0.7/89.6) at 80 °C, then cooled to 30 °C and homogenized with TG in an automatic homogenizing system (1400 rpm). Subsequently, an aqueous solution of preservatives, a solution of lecithin in propylene glycol, were mixed thoroughly with gel bases. Next, the ricin oil was carefully emulsified with the base. ALA was then dispersed in propylene glycol and homogenized with emulgel with the final concentration of 5% (*w*/*w*). Emulgels F1–F3’s composition is shown in Table 1. Drug-free emulgel bases P1–P3 (placebo formulations) were additionally prepared for rheological, mucoretention and cytotoxicity studies. All formulations were kept in closed containers at 4 ± 2 °C. The pH was assessed by pH-meter Orion 3 Star (Thermo Scientific, Waltham, MA, USA). ALA content analysis was performed with reverse-phase high-pressure liquid chromatography system (ProStar, Varian Inc., Palo Alto, CA, USA) according to [28].

### 3.3. Rheological Measurements

The rheological analysis was carried out with a Brookfield viscometer (RVDV-III Ultra, Brookfield Engineering Laboratories, Middleboro, MA, USA) equipped with a CPA52Z cone at 37 ± 1 °C. The shear rate was 2–12 s^−1^ and shearing time was 30 s. Drug-loaded and placebo emulgels were examined upon dilution with SSF in a ratio of 1:0.5 *w*/*w*, imitating the conditions upon constant saliva flow. The measurements were performed in triplicate.

### 3.4. Mucoretention Measurements

**Figure 6:**
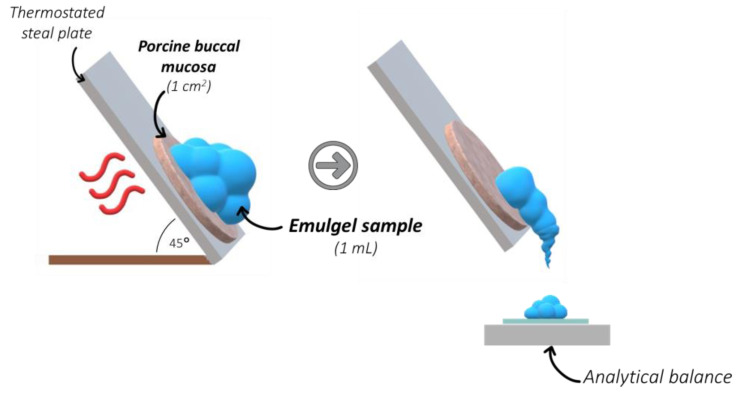
The scheme of the mucoretention test using thermostated inclined steel plate (modified according to [13]).

To examine mucoretention characteristics of the emulgels formulations, the studies were performed with freshly excised porcine buccal tissue attached to a self-constructed inclined steel plate at 36 ± 2 °C in accordance with [13,21]. The kinetic detachment was evaluated from the delay in the sample slipping, and in its complete detachment from epithelium. Several variants were examined: emulgels without dilution and upon dilution with SSF in a ratio 1:0.25 and 1:0.5 *w*/*w*, imitating the conditions upon constant saliva flow and gradual dilution of formulation [29]. Sample (1 mL) was spread thoroughly over the porcine buccal tissue (with area 1 cm^2^) fixed to horizontally positioned plate, which was set at a 45° inclination, simulating changing tongue or mouth positions (Figure 6).

The amount of sample separated from the tissue was weighed at predetermined time intervals: 2, 5, 10 and 30 min. The measurements were performed in triplicate. The mucoretention, expressed as the percentage of the sample adhered to the tissue, was calculated as follows:

Mucoretention = (W_0_ − W_t_)/W_0_ × 100 (1)

where:

W_0_—initial sample weight applied on the tissue,
W_t_—weight of the sample detached from the tissue at predetermined time point.

### 3.5. Cytotoxicity Profile

#### 3.5.1. Cells

Human primary gingival fibroblasts (PCS-201-018) and human primary gingival keratinocytes (PCS-200-014) were obtained from ATCC (Manassas, VA, USA). Fibroblasts were cultured with DMEM supplemented with 10% (*v*/*v*) FBS, 100 U/mL penicillin, and 100 μg/mL streptomycin, while keratinocytes were cultured with Dermal Cell Basal Medium (ATCC^®^ PCS-200-030TM) with Keratinocyte Growth Kit (ATCC^®^ PCS-200-040TM). Cells were seeded at a density of 10,000 cell/cm^2^ in 150 cm^2^ Corning^®^ cell culture flasks and grown at 37 ± 0.5 °C with 5% CO_2_. Cell viability was measured after each collection using optical microscopy with Trypan Blue staining. In all experiments, the cell viability was >96%.

#### 3.5.2. Sample Dilutions for Cytotoxicity Studies

Concentrated stock solutions of drug-free and ALA-loaded emulgels were prepared in sterile culture medium under an aseptic environment in a laminar flow cabinet, Lamil Plus 13 (Karstulan Metalli Oy, Finland). For each assay, the proper amount of prepared sample was added to each well (to give final concentrations of 0.01, 0.1, 0.3 or 1 mg/mL) and incubated for 4 or 24 h at 37 ± 1 °C in a 5% CO_2_ humidified atmosphere. As an additional control, pure ALA dissolved in culture medium (at final concentration 0.05 mg/mL, corresponding to the amount of drug encapsulated in formulations tested in the highest concentration, 1.0 mg/mL) was employed concomitantly with experimental emulgel samples.

#### 3.5.3. MTT Assay

A cell viability assay was performed using tetrazolium salt (MTT). After 24 h of incubation with the formulations, the culture medium was removed and the cells were rinsed with PBS (pH 7.4) in triplicate. Then, the cells were exposed to 5 mg/mL MTT solution for 20 min. After discarding the medium, a mixture of 200 μL of DMSO with 20 μL of Sorensen buffer (0.1 mol/L glycine with 0.1 mol/L NaCl equilibrated to pH 10.5) was added to each well. The absorbance was measured spectrophotometrically with an Infinite M200 PRO Multimode Microplate Reader (Tecan Group Ltd., Männedorf, Switzerland) at 570 nm. Values were presented as a percent of control cells not exposed to formulations [30].

#### 3.5.4. RealTime-Glo^TM^ Annexin V Assay

The RealTime-Glo^TM^ Annexin V Apoptosis and Necrosis Assay Kit (Promega, Madison, WI, USA) measures the exposure of phosphatidylserine on the outer surface of the cell membrane during apoptosis. Drug-free or ALA-loaded formulations were added to cultured cells seeded in 96-well plates with a density of 2000 cells/well and incubated at 37 °C, 5% CO_2_ for 24 h. Detection of apoptosis and necrosis was performed according to manufacturer instructions using 50 mM H_2_O_2_ as the positive control.

#### 3.5.5. JC-1 Assay

Mitochondrial membrane potential (ΔΨM) was investigated using tetraethylbenzimidazolylcarbocyanine iodide (JC-1), which accumulates in energized mitochondria. For this purpose, Mitochondrial Membrane Potential Assay Kit (ab113850, Abcam plc, Cambridge, UK) was used according to the manufacturer’s instructions. The insensitivity of the luminescence of cells with disrupted MMB was measured after 24 h incubation with emulgel samples and controls.

### 3.6. Statistical Analysis

The quantitative variables were expressed as the mean ± standard deviation (SD) by MS Excel software. GraphPad Prism 9.5.1 software (GraphPad Software, Inc., La Jolla, CA, USA) processed the statistical data for the cytotoxicity studies. The distribution normality was checked using the Shapiro–Wilk test, and the homogeneity of variance was evaluated using Levene’s test. A two-way ANOVA with factors including concentration and formulation was used for comparisons. The measurements were considered significant at *p* < 0.05.

## 4. Conclusions

The presented findings showed the potential of emulgels composed of mucoadhesive gelling agents as oromucosal platforms for ALA delivery. The designed formulations possessed non-Newtonian shear-thinning properties, which should enhance their spreadability over all oromucosal surfaces. The presence of xanthan gum favored the thixotropic behavior of tragacanth-based (F1) and gellan-based formulations (F3), which, in turn, would help to maintain the drug at the mucosal tissue. This work also provides insight toward understanding the influence of dilution with saliva on the mucoadhesive behavior of the tested oromucosal preparations. The combination of xanthan and gellan gums enhanced emulgel retention by porcine buccal tissue even upon dilution with saliva. In turn, the presence of delta-aminolevulinic acid was found to favor interactions with mucosal tissue, particularly formulations comprised of tragacanth. Overall, the designed formulations displayed no significant impact on cell viability after 24 h incubation in the concentration range 0.01–0.3 mg/mL. The highest tested concentration of emulgels affected cell viability by inducing an apoptotic response mediated through the mitochondrial pathway. Cytotoxicity studies also demonstrated that tragacanth-based and gellan/xanthan-based emulgels might exert a protective effect toward the metabolic activity of human gingival fibroblasts and keratinocytes. Overall, tragacanth/xanthan (F1) and gellan/xanthan (F2) emulgels with favorable thixotropic and mucoretention characteristics appeared as promising oromucosal platforms for ALA delivery. Further in vivo studies will carefully evaluate their safety profile and therapeutic efficacy toward oral lichen planus.

## 5. Patents

Szymańska et al. (2023), Patent Application Number P.443813 (PL).

## Figures and Tables

**Table 1:** Composition of ALA-loaded emulgels F1–F3.

	F1	F2	F3
Compound	Concentration (%, *w*/*w*)		
5-aminolevulinic acid	5.0	5.0	5.0
Tragacanth gum	5.0	5.0	-
Xanthan gum	1.0	-	2.0
Gellan gum	-	-	0.7
Castor oil	2.0	2.0	2.0
Lecithin	0.5	0.5	0.5
Propylene glycol	5.0	5.0	5.0
Disodium dihydrogen ethylenediaminetetraacetate	0.1	0.1	0.1
Sodium benzoate	0.1	0.1	0.1

## Data Availability

The data presented in this study are available on request from the corresponding author.
